# Synthesized Multiple Antigenic Polypeptide Vaccine Based on B-Cell Epitopes of Human Heparanase Could Elicit a Potent Antimetastatic Effect on Human Hepatocellular Carcinoma *In Vivo*

**DOI:** 10.1371/journal.pone.0052940

**Published:** 2013-01-07

**Authors:** Jun Zhang, Jian-min Yang, Hui-ju Wang, Guo-qing Ru, Dai-ming Fan

**Affiliations:** 1 State Key Laboratory of Cancer Biology and Institute of Digestive Diseases, Xijing Hospital of Digestive Diseases, Fourth Military Medical University, Xi'an, Shanxi Province, People's Republic of China; 2 Department of Gastroenterology, Zhejiang Provincial People's Hospital, Hangzhou, Zhejiang Province, People's Republic of China; 3 Key Laboratory of Gastroenterology of Zhejiang Province, Hangzhou, Zhejiang Province, People's Republic of China; 4 Department of Pathology, Zhejiang Provincial People's Hospital, Hangzhou, Zhejiang Province, China; Enzo Life Sciences, Inc., United States of America

## Abstract

**Aims:**

The aim of this study was to investigate the antimetastatic effect of multiple antigenic polypeptide (MAP) vaccine based on B-cell epitopes of heparanase (HPSE) on human hepatocellular carcinoma (HCC) in vivo.

**Methods:**

The antiserum against B-cell epitopes of HPSE was isolated, purified and characterized after immunizing white-hair-black-eye (WHBY) rabbit with freshly synthesized MAP vaccine. Tumor-bearing murine models of orthotopic implants using HCC-97H cell line were built in BALB/c nude mice. Anti-MAP polyclonal antibodies induced by MAP vaccine were administrated to the models. The impact on metastasis was assessed, the expressions of VEGF/bFGF in hepatoma tissues and in murine sera were evaluated, and the micro-vessel density (MVD) was counted as well. In addition, the possible impairments of the HPSE MAP vaccine on certain HPSE positive normal organs and blood cells were investigated.

**Results:**

The antiserum was harvested, purified and identified. The antibodies induced by MAP vaccine could specifically react with the dominant epitopes of both precursor protein and large subunit monomer of HPSE, markedly decrease HPSE activity, suppress the expressions of both VEGF and bFGF, and reduce the MVD. Pulmonary metastasis was also attenuated significantly by the anti-MAP polyclonal antibodies. In addition, no obvious impairment could be observed in certain HPSE positive organs and cells.

**Conclusion:**

MAP vaccine based on B-cell epitopes of HPSE is capable of alleviating HCC metastasis in vivo, mainly through inhibiting the HPSE activity and tumor associated angiogenesis, by virtue of the specific anti-MAP polyclonal antibodies. Furthermore, these HPSE-specific antibodies do not cause obvious abnormalities on certain HPSE positive blood cells and organs. Our study provides theoretical evidences for the clinical use of the synthesized MAP vaccine based on B-cell epitopes of HPSE in preventing HCC metastasis.

## Introduction

Hepatocellular carcinoma (HCC) is the third leading cause of cancerous deaths in the world with killing over 600,000 sufferers annually [1]. Liver transplantation and tumor resection have been proved to be the most effective standard therapies [2], and radiofrequency ablation and transarterial chemoembolization are the next preferred lines of treatment [2], [3]. Nevertheless, these therapeutic approaches usually could not offer a complete cure, as about 50% of the treated patients experience relapse within 3 years [3]. Metastasis is usually the main cause in HCC recurrence, and lungs are the most common metastatic spots [4]. Thus, it is extremely necessary to establish a complementary remedy in preventing and treating HCC metastasis.

Tumor growth, invasion and its metastasis are closely correlated with angiogenesis, which is defined as new blood capillaries engendered from pre-existing microvessels and venules [5]. Vascular endothelial growth factor (VEGF) and basic fibroblastic growth factor (bFGF) have been proposed to be the crucial endogenous factors. They have a stimulative effect on angiogenesis, causing a series of signal transduction which induces endothelial cell (EC) proliferation and promotes EC migration. All these actions ultimately lead to neovascularization [6], [7]. Microvessel density (MVD) is considered as golden standard in assessing tumor angiogenesis, and markers such as Factor VIII, CD31 and CD34 have been used in exhibiting MVD [8].

At the early stage of angiogenesis, EC sprouting relies mainly upon the enzymatic degradation of extracellular matrix (ECM) [9]. The tumor progressive cascades are also mediated by the degradation of ECM and basement membrane (BM), which allows malignant cells to penetrate through tissue barrier. Up to now, Heparanase (HPSE) is the only endoglycosidase found that can specifically degrade the heparan sulfate (HS) side chain of heparan sulfate proteoglycans (HSPG) in ECM or at BM, resulting in destructing ECM or BM, releasing multiple kinds of cytokines and facilitating cellular movements [10]. Some investigations have proved that HPSE is overexpressed in most malignancies, including in HCC, and plays a key role in cancer invasion and metastasis [10]. While HPSE is expressed at a relatively low level in mammalian lymphoid organs, leukocytes and platelets, and it is either not expressed or expressed at very low levels in other normal tissues [11], [12]. Recently, it was discovered that HPSE inhibitors could effectively suppress the invasion and metastasis of some malignant tumors [13]–[16]. Therefore, HPSE could be regarded as an important tumor associated antigen (TAA) as well as a target molecule in antitumor treatment [10], [13]–[16]. The HPSE precursor protein has a molecular weight of about 65 kDa. It is a hetero dimmer consisting of two subunits, with a molecular weight of 50 and 8 kDa, and the larger one represents the mature activated form of HPSE [17]. On the basis of human HPSE protein structure and its predicted B-lymphocyte epitopes via bioinformatics, we had designed and synthesized the multiple antigenic peptides (MAP) vaccine in our previous studies, and validated that its polyclonal antibodies had an anti-invasion potency on HCCLM6 cell lines in vitro [18]–[19].

In this study, to investigate the in vivo immune impact on human HCC metastasis, purified antibodies induced by the B-cell MAP vaccine were administrated to tumor-bearing BALB/c nude mice through passive immunity. The results showed that the MAP vaccine could elicit a potent antimetastatic effect in vivo, by virtue of its anti-MAP polyclonal antibodies. Our study probably provide new insights into the immunological prevention of HCC metastasis.

## Materials and Methods

### Ethics Statement

#### Experimental animals and cell line

Pathogen-free male BALB/c nude mice (weighing 20±2 g, SPF grade, certificate SCXK20080115) of 4 week old were purchased from Shanghai SLAC Laboratory Animal CO. LTD (Shanghai, China). White-hair–black-eye (WHBY) rabbits, derived from Japanese big-ear white rabbits, were supplied by Animal Experimental Center of Zhejiang Chinese Medical University (Hangzhou, China). All Animals were maintained at the Animal Research Center of Zhejiang Chinese Medical University. This study was approved by the ‘Medical Research Animal Ethics Committee’ (Permit Number: X1002623) of Zhejiang Chinese Medical University, and the animals were kept and the experiments were performed in accordance with committee's criteria for the care and use of laboratory animals. All surgery was performed under sodium pentobarbital anesthesia, and all efforts were made to minimize suffering. The animals were provided with water and food *ad libitum*, and quarantined under a 12 h light∶12 h dark photoperiod. The animals were acclimated for at least 1 week before any experiments were conducted. At the end of the experiments, all animals were first anesthetized (the rabbits were intravenously injected 3% pentobarbital sodium at 30 mg/kg, while the mice were intraperitoneally administrated 0.3% pentobarbital sodium at 60 mg/kg), and were euthanized by ‘acute massive blood loss’. The HCC cell line HCC97-H (HPSE positive) was purchased (Liver Cancer Institute of Zhongshan Hospital, Fudan University, China), maintained in our laboratory and routinely cultured in Dulbecco's modified Eagle's medium (DMEM) supplemented with penicillin (100 U/ml), streptomycin (100 µg/ml) and 10% fetal bovine serum (FBS). The cell line was kept at 37°C in a humidified atmosphere of 5% CO_2_.

### Preparation of B-cell MAP Vaccine of HPSE

B-cell MAP vaccine of HPSE was synthesized, purified, and identified as described previously [18]–[19]. Briefly, based on the amino acid sequence of human HPSE, we had previously selected peptide HCTNTDNPRYKEGDL (279–293) of HPSE as B lymphocytic epitopes [19], and the MAP polypeptides were synthesized by Chinese Peptide Company (Hangzhou, China) with the Solid-Phase Peptide Synthesizer by adopting eight-branched peptide design. The peptides were purified using reverse-phase high-performance liquid chromatography (HPLC) on a Vydac C18 column. The purity of the peptides was confirmed by analytic HPLC, and the MAP identity had already been confirmed in our previous investigations by binding affinity test between the MAP polypeptide and commercialized HPSE antibody, using indirect enzyme-linked immunosorbent assay (ELISA) [18]–[19].

### Animal Immunization and Isolation of Antiserum

To harvest the antiserum that containing specific polyclonal antibodies against the synthesized MAP vaccine, WHBY rabbit was immunized with the vaccine and the antiserum was isolated and identified referred to the procedure we described previously [18]. Briefly, eight-branched B-cell MAP vaccine were used to immunize WHBY rabbit intravenously for 4 times, with an interval of 2 weeks. Freund's complete adjuvant (Sigma-Aldrich, USA) was used in the first immunization, while Freund's incomplete adjuvant (Sigma-Aldrich, USA) and Th linear peptide (Chinese Peptide Company, China) were used to strengthen the immunization. Blood samples were taken before the first immunization and 10 days after each immunization, 5 times in total. The samples were taken from the central artery of the rabbits' ears at the first 4 times and taken from the carotid artery at the final time. A standardized indirect ELISA (InSight Bio Ltd, Israel) was performed to determine the specific antibody titers in the samples. WHBY rabbit was sacrificed, the blood was collected and the antiserum was separated. The anti-MAP polyclonal antibodies contained in the immunized rabbit serum were purified by caprylic acid/ammonium sulfate (CA-AS) precipitation, which is a classical, efficient and low-cost method for purification of humoral antibodies [20]. The quantity of the antibodies was determined using Coomassie Brilliant Blue (Boster Biotechnology, China) as described elsewhere [21].

### Western Blot Assay

To verify the specificity of the immunized serum, western blot assay was carried out referred to the Western blot detection kit (KangChen Bio-Tech Inc, China) and the steps we described elsewhere [18]–[19]. Certain immunoreactive bands would appear so long as the anti-MAP polyclonal antibodies contained in the antiserum could specifically react with the HPSE of the HCC97-H cells. Briefly, the proteins of HCC97-H cells were extracted shortly after the cells (3×10^7^) were lysed, and an SDS-PAGE was performed. The primary antibody was the commercialized rabbit anti human HPSE antibody (InSight Biopharmaceuticals Ltd, Israel) with a dilution of 1∶200, or the purified rabbit anti-MAP antiserum with a dilution of 1∶3000. The pre-immunized rabbit serum was used as a negative control. Immunoreactive bands were detected using chemiluminescence.

### Tumor Cell Inoculation and in situ HCC Implanting

The human HCC97-H cell line was continuously cultured. Subculture was executed every 5–6 days and the cells grew well along the walls of culture flasks. Then, the cells (1×10^7^ cells/mouse) at logarithmic growth period were subcutaneously inoculated into the right flank of 10 BALB/c nude mice. About 8 weeks later, the inoculated cells in 4 mice successfully grew up into solid tumors at approximately 1 cm^3^ while others failed in proliferation. Then the mice were euthanized and the tumors were entirely gouged out and were immediately put into saline containing 100 u/ml penicillin and streptomycin. Tumor pieces at the size of 1–2 mm^3^ were sheared from the margins of the whole mass after winkling the connective tissue. Then the tumor pieces were subcutaneously transplanted into the dorsal skin in 10 BALB/c nude mice. Six weeks post extensive subcutaneous re-transplatation, the mice were killed and the small tumor pieces were harvested using the same method outlined above. Then 30 nude mice were randomly divided into 3 groups as low-dosage antibody (LDA) group, high-dosage antibody (HDA) group, and control group, with 10 mice in each. The tumor pieces were implanted into the left lobe of the murine liver at the specific pathogen-free animal operating room of the Animal Experimental Center. Briefly, the surgical procedures were as follows. (1) The animal was first anesthetized using 0.3% pentobarbital sodium at 60 mg/kg as mentioned above; (2) Disinfection of the abdomen was performed using iodine tincture in supine position; (3) An aseptic scarf with a hole was covered onto the abdomen and a left subcostal incision of about 1.5 cm was made; (4) Left lobe of the murine liver was gently dragged out, on which an oblique incision of 0.3 cm was cautiously made and compression hemostasis was applied immediately. (5) Two tumor pieces were embedded into the small incision on the left liver lobe. (6) The left lobe was compressed for a while and was softly send back into the peritoneal cavity. (7) Finally, peritoneum, muscles and skin were sutured orderly.

### Passive Immunization in Tumor-bearing Murine Models

Four weeks after the in situ operation, B ultrasonography with ultrasonic elasto-test (Aplio XG, TOSHIBA) was carried out to confirm the existence of the implanted tumors and measure their sizes. Mean tumor volume was measured and calculated according to the formula v = ab^2^/2 (‘a’ represents the maximum diameter of the tumor, and ‘b’ represents the vertical diameter of the maximum diameter). When the in vivo models of orthotopic implants were built and validated, vaccine-derived polyclonal antibodies at the volume of 0.2 ml were used to immunize the mice in LDA group and HDA group with the antibody dosage of 2 mg/kg and 4 mg/kg, respectively, by caudal vein injection for 2 times with an interval of 2 weeks. For the control group, 0.2 ml unimmunized rabbit serum with a dilution of 1∶100 was intravenously administrated.

### Immunohistochemistry Staining

All mice were sacrificed 8 weeks after the first immunization, the implanted hepatoma was entirely excised and formalin fixed. Histological sections of the HCC were manufactured and immunohistochemically stained for VEGF, bFGF and CD34 in accordance with the instructions of the kits (YKBIOTECH, China). Immunohistochemical results of VEGF and bFGF were evaluated referring to the method we described elsewhere [19]. Briefly, the determination of experimental results was blindly analyzed by 2 senior pathological doctors. It was carried out firstly under low power lens to select the densely stained area, then 500 tumor cells were counted by the pathologists within 5 fields of 200× visual sight. The scoring standards of immunohistochemistry staining were based on the coloring of the cancer cells and on the percentage of positive cells: I (color): no staining: scores 0, light yellow: scores 1, brown: scores 2, dark brown: scores 3; II (percentage): negative: scores 0, the percentage of positive cells ≤10%: scores 1, 11–50%: scores 2, ≥51%: scores 3. While the intensity of VEGF or bFGF expression was denoted by the product of cancer cells staining intensity and positive cell percentage: 0–2: scores −, 3–4: scores +, 5–7: scores ++, and 8–9: scores +++.

A single microvessel was defined as any brown or brownish yellow CD34-immunostained endothelial cell, which was used in reflecting MVD [8]. We evaluated MVD following the method mentioned by Weider [8]: high vascular density area was selected under low power objective, and the numbers of vascular stained by CD34 were counted in 3 visual fields under high-power microscope (400×), and the average value was regarded as the MVD value of the tumor.

### ELISA for VEGF and bFGF in Murine Serum

Blood samples were drawn from the murine orbits before executing. Sera were separated using centrifuge and were assayed in ELISA cultures (96-well coated microtiter plates; DAKEWE) for VEGF or bFGF concentration, and procedures were referred to the user manual of the ELISA kit (CUSABIO, Wuhan). Briefly, the steps were as follows. (1) 100 µl of Standard, Blank or Sample was plated in triplicate per well in a 96-well plate and incubated for 2 hours at 37°C; (2) 100 µl of Biotin-antibody working solution was added to each well and incubated for 1 hour at 37°C; (3) Each well was aspirated and washed, then the above-outlined process was repeated 3 times for a total of 3 washes; (4) 100 µl of HRP-avidin (Horseradish peroxidase-avidin) working solution was added to each well and incubated for 1 hour at 37°C; (5)The aspiration and wash were repeated 5 times; (6) 90 µl of TMB (3,3′,5,5′-Tetramethylbenzidine) Substrate was added to each well and the plate was incubated for 15–30 min at 37°C; (7) 50 µl of Stop Solution was added to each well when the first 4 wells which containing the highest concentration of standards developed obvious blue color; (8) The optical density of each well was determined using a microplate reader set to 450 nm. Accordingly, a standard curve could be depicted using the professional soft “Curve Exert 1.3” and the VEGF or bFGF concentration could be thus reckoned.

### Assessment of Pulmonary Metastasis

Shortly after all mice were euthanized, lungs were totally excised and formalin fixed, and histological slices were manufactured from 5 coronal sections of the lungs. In every pulmonary section, five slices at 4∼5 µm in thickness were continuously made with the interspace of 0.8 mm. Then H&E staining was performed and pulmonary metastatic micro foci were observed and counted by 2 senior pathological doctors under 100×or 200× microscopic lens. A single micro focus could be counted only once even if presented in more than one slice. The total number of metastatic foci in each group was summed up. The mean pulmonary foci number per nude mouse was calculated in order to investigate whether there were any significant differences among these groups.

### Impairments on Certain HPSE Positive Organs and Blood Cells

To preliminarily estimate the possible impairments of the B-cell MAP vaccine on certain HPSE positive organs and cells, cervical lymph nodes of the rabbit and spleens of all the animals were resected and formalin fixed shortly after the animals were sacrificed. Histological sections of the spleens and lymph nodes were made and H&E staining was performed in order to detect the possible pathological changes. The amounts of certain HPSE positive blood cells such as lymphocytes, neutrophils and platelets were counted. Peripheral blood smears were manufactured and possible morphological changes of lymphocyte, neutrophil and platelet were observed under 1000× high-power lens.

### Statistical Analysis

Mean ± SD was calculated from the experimental results. Statistical analysis was conducted using Student's t test or One-way ANOVA Analysis. Statistical significance was defined as p<0.05. All statistical analyses were conducted using SPSS 11.5 software (SPSS Inc., Chicago, Illinois, USA).

## Results

### Harvest of The Immunized Rabbit Antiserum

The identity of the synthesized MAP polypeptide had already been confirmed in our previous investigations and the purity of the MAP polypeptides could reach 97% or more by purification through HPLC [18], [19]. Ten days after the first active immunity in WHBY rabbits using B-lymphocyte MAP vaccine of human HPSE, specific antibodies against the MAP vaccine could be detected in its serum. The highest specific antibody titer after immunization could reach 1∶140,000. The isolated antiserum was purified successfully by CA-AS precipitation, and the concentration of the anti-MAP polyclonal antibodies contained in the purified antiserum was 15.2 mg/ml, determined by Coomassie Brilliant Blue assay.

### Western Blot Assay

**Figure 1:**
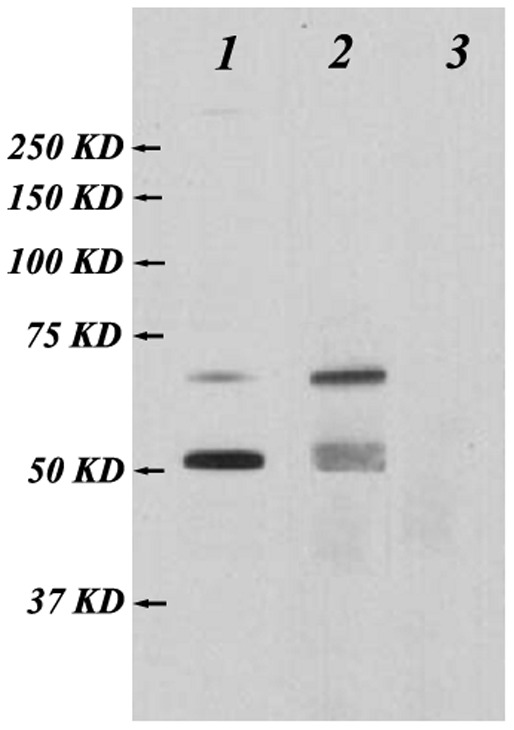
Specificity of the anti-MAP polyclonal antibodies contained in the rabbit antiserum measured by western blot analysis. The total protein extracted from the HCC97-H cells was used to react with the anti-MAP polyclonal antibodies. The commercialized polyclonal rabbit anti-human HPSE antibody was used as positive control and the corresponding rabbit pre-immunized serum was used as negative control. A very clear band of about 50 kDa and a clear band of about 65 kDa are recognized in column 1, using the commercialized heparanase antibody (1∶200). Both 65 kDa and 50 kDa bands are exhibited in column 2, using the anti-MAP antiserum (1∶3000). No band is detected in the corresponding locations in column 3, using unimmunized rabbit serum (1∶3000).

The total protein extracted from the HCC97-H cells was used to react with the anti-MAP polyclonal antibodies. The commercialized polyclonal rabbit anti-human HPSE antibody was used as positive control and the corresponding rabbit pre-immunized serum was used as negative control. Chemiluminescence showed a very clear band of about 50 kDa and a clear band of about 65 kDa in the commercialized antibody group. In the anti-MAP antiserum group, both 65 kDa and 50 kDa bands were exhibited distinctly. According to the instructions of the commercialized antibody, the protein of 65 kDa is most probably the precursor HPSE protein of HCC97-H cells, while the 50 kDa protein corresponds to its large subunit. In contrast, no band could be detected in the corresponding locations for the negative control (Fig. 1).

### Tumor Volume 4 Weeks after The in situ Implanting Operation

**Figure 2:**
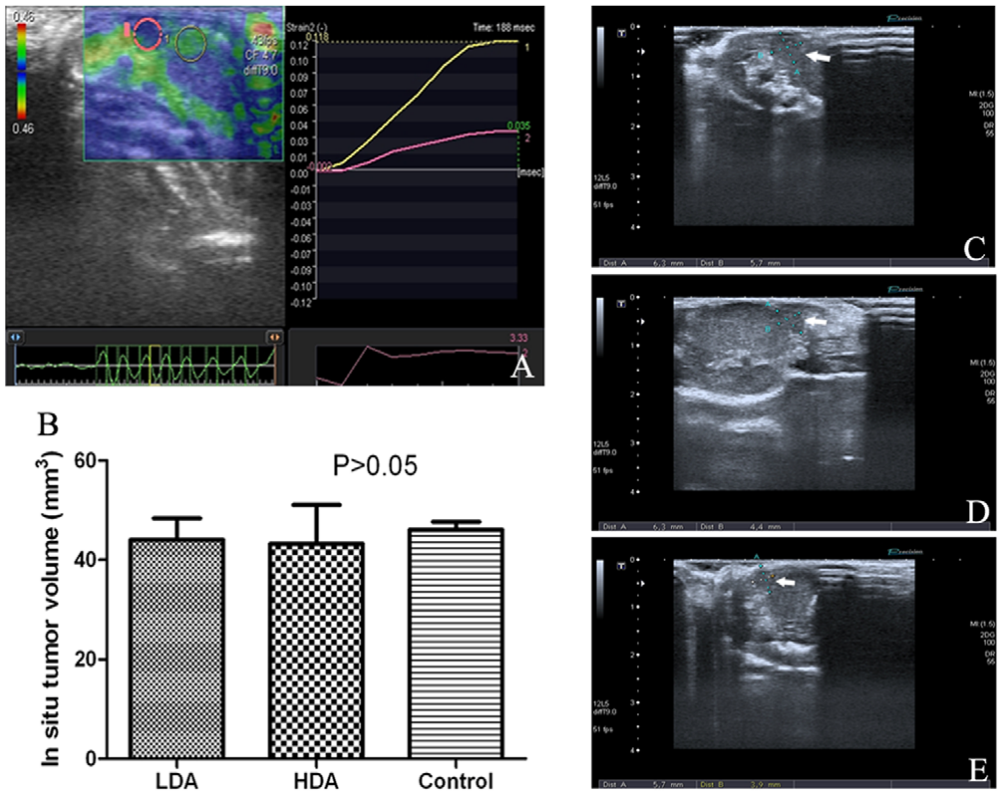
Representative images of ultrasonography. B ultrasound scan with ultrasonic elasto-test was carried out 4 weeks after the in situ implantation. The elasticity of the low-echo hepatic region (within the pink circle) was much lower than that of the normal-echo hepatic region (within the yellow circle), as shown in picture A. Thus, it could be deduced that the low-echo region should be the in situ implanted malignancy. Picture C, D and E are representative ultrasound images of LDA group, HDA group and control group, respectively. Figure B shows there was no statistical difference in mean tumor volume among each group (44.03±7.51 mm^3^, 43.24±13.55 mm^3^, and 46.07±4.57 mm^3^, respectively, P>0.05).

In situ HCC-implanting operation was carried out successfully and no mouse was dead during the surgery. B ultrasonography with ultrasonic elasto-test was carried out at the end of the 4th week after this operation. Representative image of the ultrasonic elasto-test was shown in Figure 2A, which confirmed that the low-echo area in murine liver was the implanted malignancy. Representative ultrasonic images of LDA group, HDA group and control group are shown in Figure 2C–E. Several in situ tumor pieces failed in growth or even vanished, thus these mice were excluded from this study and the final amount of the mice in LDA group, HDA group and control group were 8, 8 and 9, respectively. There was no statistical difference in mean tumor volume among each group (P>0.05, Figure 2B).

### Expression of VEGF, bFGF and CD34 in The Implanted Hepatoma

**Figure 3:**
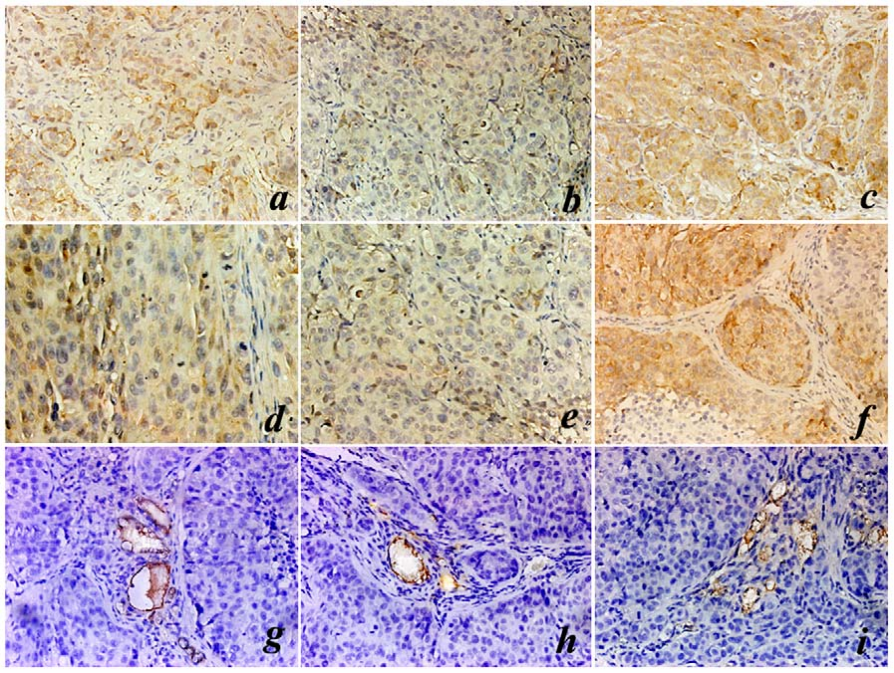
Representative images of immunohistochemistry staining. Eight weeks after the first passive immunization, mice were sacrificed and immunohistochemistry staining of VEGF, bFGF and CD34 (MVD) on the in situ HCC tissues were performed. (a–c) representative VEGF immunostaining images of LDA group, HDA group and control group, respectively (400×); (d–f) representative bFGF immunostaining images of LDA group, HDA group and control group, respectively (400×); (g–i) representative CD34 immunostaining images of LDA group, HDA group and control group, respectively (400×).

All tumor-bearing mice survived till the end of the experiment (8 weeks after the first passive immunization). Immunohistochemical staining of VEGF and bFGF were performed on the orthotopic hepatoma samples. According to the scoring standard of the immunostaining, the expressions of either VEGF or bFGF in LDA group, HDA group and control group were ++, + and +++, respectively (Table 1). While the mean value of MVD (represented by CD34 immunostaining) in sections of LDA group, HDA group and control group was 18.33±4.51/field, 10.67±4.04/field and 28.33±5.51/field, respectively. There were statistical differences among each group (P<0.05). The values of MVD are also summarized in Table 1. Representative immunostaining images of VEGF, bFGF and CD34 are illustrated in Figure 3.

**Table 1:** Expression of VEGF, bFGF and the MVD in HCC tissues.

	VEGF	bFGF	MVD
LDA group	++	++	18.33±4.51^*,**^
HDA group	+	+	10.67±4.04*
control group	+++	+++	28.33±5.51

VEGF or bFGF lowly expressed (+) in HDA group (4 mg/kg antibody), moderately expressed (++) in LDA group (2 mg/kg antibody) and strongly expressed (+++) in control group (pre-immunized rabbit serum). MVD count in either LDA group or HDA group was significantly fewer than that in control group (*P<0.05, as compared with control group), and there was also statistical difference between LDA group and HDA group (**P<0.05, as compared with HDA group).

### Concentrations of VEGF and bFGF in Murine Sera

**Figure 4:**
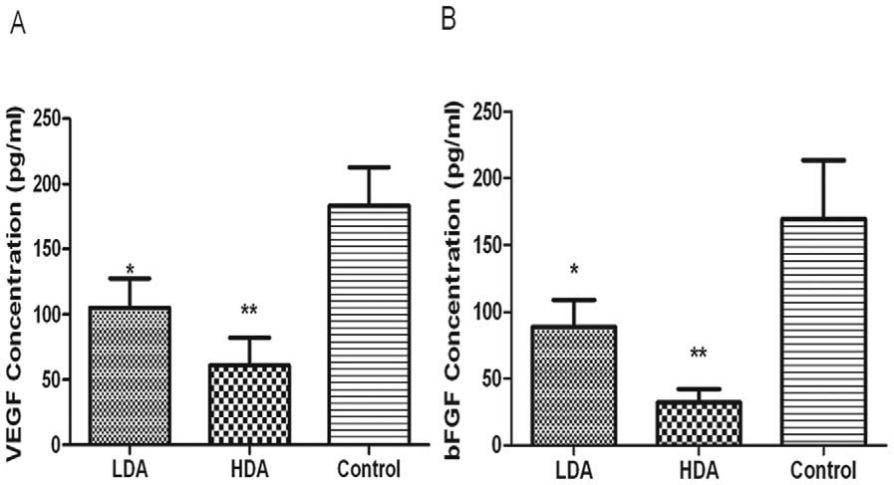
Serous concentration of VEGF and bFGF by enzyme-linked immunosorbent assay (ELISA). Blood samples were drawn from the murine orbits. Concentrations of VEGF and bFGF are illustrated in figure (A) and (B). VEGF concentrations in LDA group, HDA group and control group were 105.07±25.66 pg/ml, 61.03±21.40 pg/ml and 183.13±29.62 pg/ml, respectively. And serous bFGF levels in the 3 groups were 88.57±20.26 pg/ml, 32.13±10.09 pg/ml and 169.50±44.09 pg/ml, respectively. *P<0.05, as compared with control group. **P<0.01, as compared with either LDA group or control group.

To quantitatively evaluate the impacts of the polyclonal anti-MAP antibodies on serous concentration of VEGF and bFGF, which were released mostly by HPSE enzymolysis [10], [11], we analyzed the serum VEGF and bFGF level by ELISA as well. The mean VEGF concentrations in LDA group, HDA group and control group were 105.07±25.66 pg/ml, 61.03±21.40 pg/ml and 183.13±29.62 pg/ml, respectively, while bFGF levels in the corresponding groups were 88.57±20.26 pg/ml, 32.13±10.09 pg/ml and 169.50±44.09 pg/ml, respectively. The mean concentration of VEGF or bFGF in either LDA group or HDA group was much lower than that in control group, and the serous level of VEGF or bFGF in HDA group was also markedly lower than that in LDA group (P<0.01), which shows a certain dose-dependence, as shown in Figure 4.

### Pulmonary Metastatic Micro Foci

**Figure 5:**
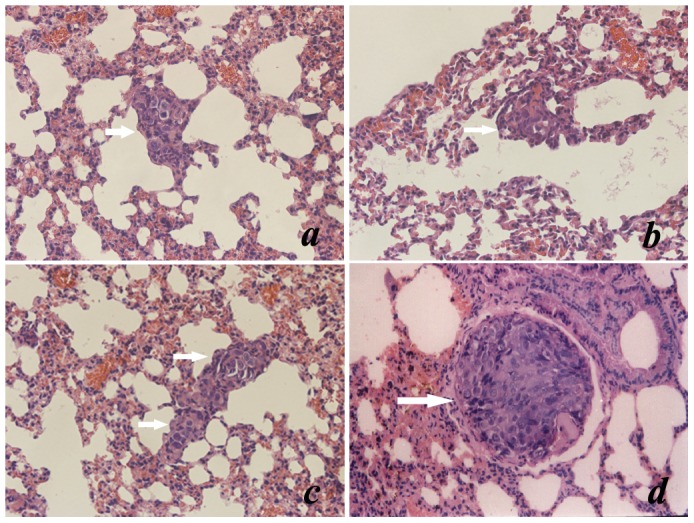
Representative images of pulmonary metastatic foci. Shortly after mice were sacrificed, lungs were entirely resected and were fixed in formalin, histological slices were manufactured. (a) and (b) are the representative H&E staining images of pulmonary metastatic foci of LDA group and HDA group, respectively (200×); (c) and (d) are the images of control group, illustrating representative pulmonary metastatic colonizations and intravascular tumor embolus, respectively (200×).

**Figure 6:**
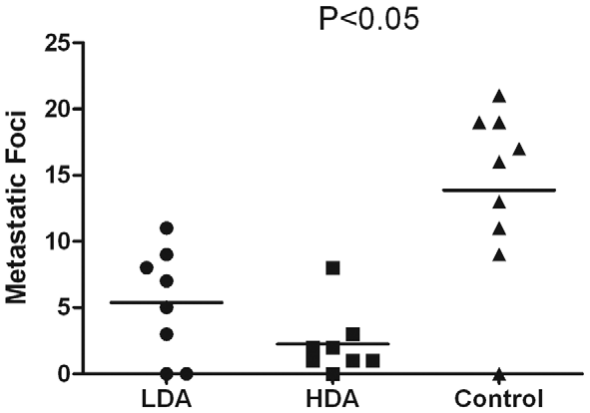
Pulmonary metastasis could be attenuated by the purified antiserum induced by B-cell MAP vaccine of human HPSE. Pulmonary micro-colonizations per mouse in LDA group, HDA group and control group were 5.38±4.10, 1.38±1.51 and 13.89±6.55, respectively. The metastatic foci in LDA group or HDA group were significantly fewer than that in control group (P<0.05). And the metastatic foci in LDA group were also markedly fewer than that in HDA group (P<0.05), which demonstrated a certain dose-dependent effect.

To determine the antimetastatic ability of the polyclonal antibodies induced by B-cell MAP vaccine of HPSE, we immunized BALB/c nude mice in LDA group and HDA group with the diluted antiserum. The unimmunized rabbit serum was administrated to the control group. All mice were sacrificed 8 weeks after the first passive immunization, and pulmonary metastatic micro foci were counted. The metastatic macro foci could not be observed with naked eyes, which could be detected under microscopy by H&E staining and were mostly located at the margins of the lungs. Representative images of pulmonary metastatic foci are shown in Figure 5. Pulmonary micro colonizations per mouse in LDA group, HDA group and control group were 5.38±4.10, 1.38±1.51 and 13.89±6.55, respectively, and there were statistical significances among each group (P<0.05), as shown in Figure 6. The metastatic foci in LDA group or HDA group were much fewer than that in control group (P<0.05). Moreover, the foci in LDA group were also markedly fewer than that in HDA group (P<0.05), which demonstrated a certain dose-dependent effect.

### HPSE Associated Impairments Could not be Detected

**Figure 7:**
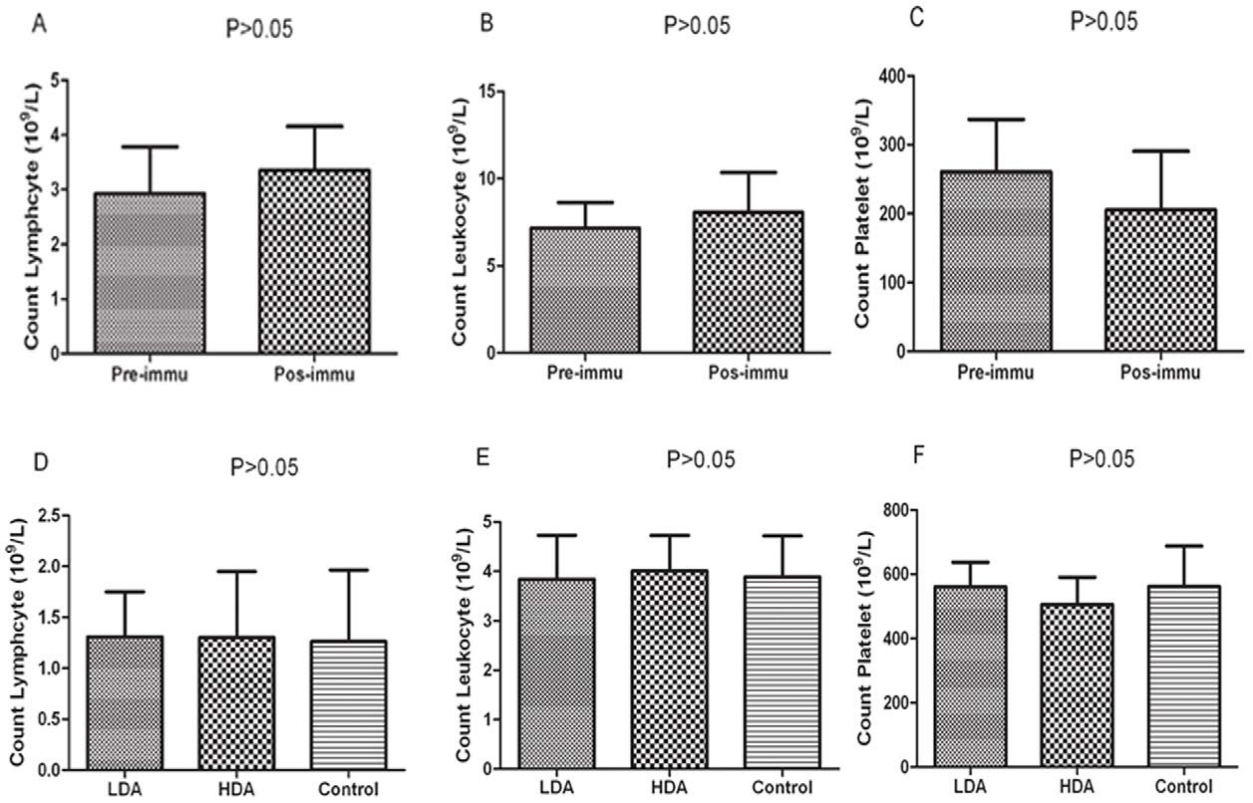
Counting blood cells of WHBY rabbit and nude mice. (A–C) The blood cells of WHBY rabbit were counted in triplicates before immunization and shortly after sacrifice, the results showed there were no significant disparities in counting lymphocytes, leukocytes and platelets (P>0.05). (D–F) Certain HPSE positive blood cells were also counted in LDA group, HDA group and control group shortly after all mice were euthanized, there were no significant differences in counting murine lymphocytes, leukocytes and platelets among these 3 groups (P>0.05).

No obvious impairments could be detected in H&E-staining pathological slices of spleens and lymph nodes. Moreover, no morphological change of rabbit or murine blood cells such as lymphocytes, neutrophils and platelets could be observed in peripheral blood smears. The rabbit blood cells were counted in triplicates before immunization and shortly after sacrifice, the results showed that there were no significant disparities in lymphocyte numbers (2.93±0.49×10^9^/L vs 3.35±0.47×10^9^/L, P>0.05), leukocyte numbers (7.17±0.83×10^9^/L vs 8.07±1.13×10^9^/L, P>0.05) and platelet numbers (2607±76×10^9^/L vs 205±85×10^9^/L, P>0.05), as shown in Figure 7A–C. Certain HPSE positive blood cells were also counted in LDA group, HDA group and control group shortly after all mice were euthanized, there were no statistical differences in murine lymphocyte numbers (1.31±0.43×10^9^/L, 1.30±0.65×10^9^/L and 1.26±0.69×10^9^/L, respectively, P>0.05), leukocyte numbers (3.84±0.89×10^9^/L, 4.01±0.72×10^9^/L and 3.89±0.83×10^9^/L, respectively, P>0.05) and platelet numbers (560±132×10^9^/L, 505±148×10^9^/L and 562±218×10^9^/L, respectively, P>0.05) among these three groups, as shown in Figure 7D–F.

## Discussion

HCC is one of the most common solid tumors in the world and its incidence is extremely high in China [22]. It has been a critical disease due to late presentation with large tumors and metastatic lesions [3]. Metastasis is the main cause of death in HCC sufferers, since it is still incurable [3], [4]. The key and necessary step in metastasis is that the tumor cells get through natural barriers composed of BM and ECM, which mainly compose of two ingredients: structural proteins and glycosaminoglycan with its main component HSPG [9]. As yet, there are as many as 17 kinds of matrix metalloproteinase (MMPs) found to be involved in the degradation of ECM [9]. Therefore, it will be difficult to suppress tumor metastasis through developing MMPs inhibitors.

However, HPSE is the sole endoglycosidase capable of degrading the ECM and BM, by spliting HS chain of glycosaminoglycan. Some studies showed that certain HPSE inhibitors, such as polysaccharides, siRNA, and polypeptide antibodies, had the potency of suppressing tumor invasion, metastasis and angiogenesis [10], [23], [24]. For example, PI-88, a member of polysaccharides, have just entered stage-III anti-tumor clinical trials [16]. For this reason, HPSE is regarded as a promising and crucial target molecule for anti-tumor investigations.

A major achievement in the field of tumor immunology over the past 20 years has been the clear demonstration that T-cell or B-cell epitopes rather than integral TAA, that induce anti-tumor immunoreactions [25]. The first group of immunogenic epitopes in HPSE had been discovered by Sommerfeldt and colleagues adopting the SYFPEITHY algorithm to identify nonapeptides of the HPSE amino acid sequence [26]. In our previous investigations, we had screened and identified that peptide fragment 279–293 of the large subunit in HPSE was a dominant B-cell epitope, and had accordingly designed and synthesized the B-cell MAP vaccine of HPSE as mentioned above. The polyclonal antibodies against such MAP vaccine could also significantly inhibit invasion capability of HCCLM6 cells in vitro in our previous studies [18], [21]. However, no research on B-cell MAP vaccine of HPSE hindering HCC metastasis in vivo has been reported till now.

In the present study, we successfully built the in situ tumor-bearing murine models using the HCC97-H cell line. This cell line had a high metastatic potential, the rate of metastasis to lung was 100% using orthotopic xenograft [27]–[29]. Nevertheless, it had a low proliferative rate, which accordingly had poor tumor-formation rate if lay simply upon subcutaneous inoculation [29]. Moreover, the transplanted tumor piece in murine liver was so small that it could be easily confused with other low-echo hepatic changes under ultrasound, such as angioma, cyst, micro vessels and bile ducts, etc [30]. Therefore, B ultrasonography with elasto-test was carried out, and it showed that the ultimate tumor-formation rate was more than 80% (25/30), and there was no statistical significance of tumor size among each group before the onset of administration. In this study, we administrated the tumor-bearing nude mice with the purified antiserum containing the anti-MAP polyclonal antibodies, to evaluate its anti-metastatic potency. And such passive immunization toward immunodeficiency genetic engineering mouse had already been approved to be safe enough and practical as early as in 1981 by Katz M et al, as long as the volume of heterogenic antiserum administrated to the mouse was no more than 0.2 ml [31]. The antibody quantity administrated to the tumor-bearing mice was consulted to the clinical usage of Herceptin in treating patients with HER2-positive tumours, which was a humanized antibody specifically developed to target human epidermal growth factor receptor 2 (HER2) [32].

To validate whether the anti-MAP polyclonal antibodies contained in the antiserum could specifically react with HPSE protein of HCC97-H cells, Western blot assay was conducted shortly after the rabbit antiserum was purified. The analysis showed 2 clear bands in anti-MAP antiserum group located at about 65 and 50 kDa. No bands appeared at the target site for pre-immunized rabbit serum. These results indicate that the antibodies induced by MAP vaccine could specifically react with the dominant epitopes of both the precursor protein and the large subunit monomer of HPSE. HPSE polypeptide antibodies can block HPSE, reduce the content of HPSE or decrease the enzymatic activity [33]. Levy-Adam et al discovered that a binding domain exists in the region of N-terminus of the HPSE 50 kDa large subunit, and the antibodies derived from the epitopes contained in that region can inhibit the HPSE activity [33]. In our previous investigations [18], [21], the HPSE activity could also be markedly decreased when treated with anti-MAP polyclonal antibodies induced by the B-cell MAP vaccine of HPSE, although our epitope (peptide fragment 279–293 of the large subunit) was not located in the N-terminus of the 50 kDa large subunit [18], [19].

To determine whether there was an anti-metastatic potency, the anti-MAP polyclonal antibodies contained in the purified antiserum were intravenously administrated to the murine models of orthotopic xenografts, while the mice in control group was given unimmunized rabbit serum. We found that pulmonary metastatic micro foci in LDA group or HDA group were significantly fewer than that in control group, which suggested that the antibodies induced by B-cell MAP vaccine of HPSE could effectively alleviate the metastasis of HCC in mice. We also discovered that there was statistical significance between LDA group and HDA group, which demonstrated that the antimetastatic effect was potent and dose-dependent.

HPSE is capable of releasing and activating HS-binding factors such as bFGF and VEGF from HSPG, which are basilic positive regulators of tumor associated angiogenesis, stimulating endothelial cell proliferation and enhance vascular permeability [5]. Recent studies reported that the angiogenic cytokines such as VEGF, bFGF were closely related to tumor growth, metastasis and the prognosis of HCC patients [34], [35]. In this study, to achieve VEGF and bFGF semi-quantitative immunological analysis, the percentage of positive staining tumor cells was calculated. The result showed that the expression of either VEGF or bFGF in LDA group and HDA group was much lower than that in control group; moreover, VEGF and bFGF expressed higher in LDA group than in HDA group. Further, we quantitatively assayed the serum VEGF and bFGF concentration, and found that the serous levels of both VEGF and bFGF in LDA group and HDA group were significantly lower than that in control group. The concentrations of VEGF and bFGF in HDA group were also significantly lower than that in LDA group, which showed a certain dose-dependence. MVD was deemed as golden standard of angiogenesis and was presented by CD34 immunostaining, in our study, we counted the mean value of MVD in the immunochemical slices of these 3 groups. The result demonstrated that MVD count in LDA group or HDA group was much fewer than that in control group. There was statistical difference between HDA group and LDA group as well, which illustrated that the effect on reducing MVD was also dose-dependent. It also showed positive correlation between VEGF/bFGF levels and MVD, that was, the stronger the expression of VEGF or bFGF, the higher the MVD, which was similar to some other researchers' findings [36]. Our results demonstrated that the synthesized B-cell MAP vaccine, which is capable of generating specific polyclonal antibodies against HPSE polypeptides through active immunization, could potently inhibit the release and the expression of VEGF and bFGF, reduce the MVD, and suppress the angiogenesis.

From a safety standpoint, the use of HPSE B-cell epitope as a TAA in vaccination may poses the risk of autoimmune side effects because HPSE is expressed not only in malignant tumors but also in some blood cells, such as T and B lymphocytes, macrophages, neutrophils, and platelets [23], [37]. We preliminarily evaluated the possible damages on certain HPSE positive organs and blood cells as well, and our results demonstrated that no obvious abnormalities could be found in counting blood cells, pathological slices of spleens or lymph nodes, and morphology of certain blood cells. However, such investigations were shallow and preliminary, other intensive experiments should be carried out to further confirm the safety of the HPSE polyclonal antibodies and the MAP vaccine.

In summary, this study suggests that the synthesized MAP vaccine based on B-cell epitopes of human HPSE is capable of attenuating human HCC metastasis in vivo, which is probably induced by suppressing HPSE activity [18], [21] and tumor associated angiogenesis, by virtue of the anti-MAP polyclonal antibodies. Furthermore, these HPSE-specific antibodies do not cause obvious impairments in certain HPSE positive immune organs and cells. Therefore, our investigations provide theoretical evidence for the clinical use of synthesized MAP vaccine based on B-cell epitopes of HPSE in preventing HCC metastasis.
