# Expression of Concern: Synthesized multiple antigenic polypeptide vaccine based on B-cell epitopes of human heparanase could elicit a potent antimetastatic effect on human hepatocellular carcinoma *in vivo*

**DOI:** 10.1371/journal.pone.0226124

**Published:** 2019-12-02

**Authors:** The PLOS ONE Editors

After publication of this article [1], concerns were raised regarding Figure 3. Specifically, there is a region of similarity between panel 3b and panel 3e, when rotated 180 degrees.

Panels 3b and 3e are reported to represent samples from the same treatment group immunostained for VEGF and bFGF, respectively, which the authors stated were generated using adjacent slices made by serial section in the same paraffin-embedded hepatoma. Editorial follow up on the concerns noted that the appearance of the VEGF staining and the bFGF staining shown in the panels 3b and 3e is highly similar. The authors provided two alternative images of replicates of bFGF immunohistochemistry in the high dosage antibody (HDA) treatment group as replacements for panel 3e. Underlying image and data files for the rest of Figure 3 and the other figures in the article are no longer available.

Evaluation of the replicate images provided for panel 3e, representing the HDA treatment group, found that one replicate contained regions of similarity when compared with the image published as panel 3a, reported to be from an animal in the low dosage antibody (LDA) treatment group.

The corresponding author acknowledges an error in image selection and placement but claims that this did not affect the statistical analyses and the final conclusions of the study.

Owing to the remaining concerns about the data reported in Figure 3, the *PLOS ONE* Editors issue this Expression of Concern.
